# The Exposed Childhood: An Examination of Chinese Parents’ Online Sharing of Children’s Photos and Videos—An Analysis Based on Douyin Network Data

**DOI:** 10.3390/bs16040499

**Published:** 2026-03-27

**Authors:** Yaping Yue, Yuang Guo, Haojie Yuan

**Affiliations:** 1 Faculty of Education, Henan University, Kaifeng 475004, China; 2 Faculty of Educational, Northeast Normal University, Changchun 130024, China

**Keywords:** children’s digital representation, parental sharenting, media spectacle, child influencer

## Abstract

Amid the prevailing trend of “pan-entertainment” in cyberspace, adults increasingly interpret children’s lives through utilitarian, adult-centric, and entertainment-focused perspectives, leading to the alienation of children’s online images. This study examines child influencer accounts on Douyin—typically managed by parents—and conducts content and discourse analysis on them. Drawing on critical theories by Douglas Kellner, we employed Scrapy and NVivo to analyze 30 popular children’s videos and 15,000 user comments posted beneath them. The analysis identifies five key characteristics in the construction of such images: spectacular visual mechanisms, younger-age production trends, covert commercial penetration, homogenized spectacle types, and adult-centric implicit influence. The study underscores the urgency of strengthening protective mechanisms to counteract platform capitalism’s intrusion into childhood and to uphold children’s digital privacy and agency.

## 1. Introduction

The rise of short video platforms has fundamentally democratized content creation, enabling individuals to produce and share videos without the need for professional equipment or formal training (Lu, 2024). A prominent manifestation of this trend in China is the widespread practice of “sharenting”—a portmanteau of sharing and parenting that refers to parents’ routine online disclosure of their children’s daily lives (Motevalli et al., 2025). According to the 52nd Statistical Report on China’s Internet Development, the number of social media users in China had exceeded one billion by June 2023. Particularly striking is that over 75% of young parents in China use parenting apps to engage in such sharing behaviors (Wang et al., 2025).

However, this participatory turn in digital media has also given rise to a pervasive “pan-entertainment” culture, a concept first articulated by Postman (1985). In his seminal work Amusing Ourselves to Death, Postman famously observed: “All public discourse increasingly takes the form of entertainment, becoming a cultural spirit. Our politics, religion, news, sports, education, and commerce have willingly become appendages of entertainment. The result is that we have become a species amused to death”. Expanding on this notion, contemporary scholars argue that the essence of pan-entertainment lies in the trivialization of serious culture through lowbrow amusement—using entertainment as a vehicle that masks underlying value erosion and nihilism, thereby gradually undermining the rational authority of mainstream ideology (Zhang, 2025). In the digital context, this phenomenon involves the strategic repackaging of diverse subjects into entertaining spectacles designed to maximize public engagement (Xun, 2024).

This notion of “spectacle” finds its theoretical elaboration in the work of French philosopher Guy Debord and American critical theorist Douglas Kellner, whose frameworks provide analytical tools for understanding how pan-entertainment operates on short video platforms. Debord (2021) posits in The Society of the Spectacle: “In modern capitalist society, authentic social relations are supplanted by representations (spectacle), where people comprehend the world through consuming media symbols rather than direct experience”. Building upon this theoretical foundation, American critical theorist Kellner (2003) advances the concept in media spectacle, defining it as “those dramatized, conflict-laden, and highly visual media events or cultural phenomena that mold social values, steer public behavior, and reinforce dominant power ideologies”. Kellner (2003) delineates five defining characteristics of media spectacles: (1) Dramatization—employing exaggerated narratives (e.g., celebrity scandals, political controversies) to capture attention; (2) Visual Spectacle—privileging sensory stimuli like imagery and short videos over substantive reflection; (3) Commodification—ultimately serving consumerist ends (e.g., influencer marketing); (4) Ideological Domination—consolidating mainstream values (e.g., success doctrine, appearance-based economy); and (5) Pseudo-Participation—creating illusions of user interaction while subjecting participants to algorithmic discipline.

These spectacle characteristics are particularly salient in the realm of children’s content, where the “pan-entertainment” logic systematically reconstructs childhood experience into consumable visual drama. Importantly, children’s content on short video platforms is not solely shaped by parental creators but emerges through a dynamic process of co-production between content creators and audiences. This co-productive dynamic reinforces the prevailing pan-entertainment logic, wherein portrayals of childhood are increasingly shaped by spectacle-driven priorities that privilege entertainment value. As Horton (2015) notes, within this paradigm, children’s life worlds are often reduced to a hybrid of pedagogical instrumentality and commercial exploitation, governed largely by adult-driven entertainment industries.

The commercial logic underlying this transformation manifests clearly in the practice of sharenting. In an increasingly commercialized digital environment, the practice of sharenting has conveniently provided opportunities for ordinary parents, who may have initially intended only to digitally archive their children’s life moments, to become influencers through applying various strategic approaches to maximizing visibility, aggregating followers, endorsing commercial brands, and eventually monetizing their children’s digital labor (van Driel & Dumitrica, 2021). An influencer is a specific type of micro-celebrity who not only gains fame through self-performance by employing digital media technologies (Senft, 2013), but possesses the power to affect the purchasing decisions among their followers. In this context, children are often commodified, with their behaviors and data exploited for commercial gain (Liu et al., 2024). As Hubbard (2002) argues, children are no longer viewed as citizens but as “miniature workers”. whose every action is mined for surplus value. Such commercial exploitation is particularly problematic given that children are inherently involved in income-generating labor processes (Kojok, 2022). Since 2021, supported by a series of favorable policies, China’s maternal and child market has expanded significantly, emerging as a sector with considerable commercial potential. This growth has further accelerated the commercialization of social media accounts that gained popularity through sharenting. Consequently, content posted by parents has increasingly shifted toward deliberately orchestrated performances by children (X. Huang, 2023).

Therefore, situated at the intersection of pan-entertainment culture, spectacle-driven media logic, and the commercialization of sharenting, this study focuses on child influencer accounts managed by parents. Drawing on the theoretical framework of media spectacle, it investigates how children’s online images are constructed within the context of pan-entertainment. Douyin was selected as the primary research site—not only because of its large user base, but also due to the platform’s embodiment of a distinctive tension in platform governance: since the introduction of age-stratified protection mechanisms in 2021, its algorithms have continued to prioritize highly entertaining children’s content alongside the implementation of these protective measures. This duality makes Douyin an ideal site for examining the construction of children’s online images under the logic of pan-entertainment.

### 1.1. Risks and Privacy Concerns in the Digital Exposure of Children

A central concern in the literature is the inherent risk of sharing children’s lives online, with “sharenting” identified as a practice that can compromise children’s privacy and long-term well-being. Early work by Choi and Lewallen (2018) issued a prescient warning that online content, once shared, could lead to negative outcomes such as criminal exploitation. This concern has been substantiated by subsequent empirical studies. For instance, Wachs et al. (2021), through a longitudinal survey of Iranian families, found a significant intergenerational effect in sharenting, noting that approximately 72% of shared content contains sensitive information. Esfandiari and Yao (2023) argued that even parents’ unconscious social sharing can cause harm, with effects potentially persisting into adulthood. To contextualize the most severe dangers, Burn (2022) highlighted that this growing phenomenon can expose children to threats such as sexual exploitation, future emotional distress, and “digital kidnapping”, even when sharenting is unintentional. Most recently, Coelho (2025) provided a comprehensive overview of sharenting’s risks to children’s privacy, safety, and psychological well-being, reinforcing the ongoing relevance of this research area.

### 1.2. The Construction of Children’s Digital Identities

Beyond the risks of exposure, a growing body of research examines how children’s digital identities are actively shaped and negotiated.

A substantial portion of the literature focuses on how parents construct their children’s digital identities. Choi and Lewallen’s (2018) content analysis of 510 Instagram photos revealed that parents’ digital portrayals, while increasing the visibility of marginalized groups, nonetheless perpetuate traditional gender and racial stereotypes. He et al. (2022) conducted a thematic analysis of stay-at-home mothers’ short videos on Douyin. They identified three distinct forms of labor in digital maternal practices—domestic, emotional, and entrepreneurial—and, based on these patterns, proposed the concept of “Motherhood 3.0” as an update to the existing “Motherhood 2.0” framework. Berg et al. (2024), through a scoping review using PRISMA-ScR guidelines, synthesized this field, identifying sharenting, digital footprints, and children’s identities as key themes. More recently, Zhao (2024) applied multimodal discourse analysis to videos by overseas Chinese parent influencers on Douyin, finding that despite differing representations of diasporic parenthood.

A more recent and directly relevant strand of research focuses on child influencers as active participants in the digital economy. Meng (2018) provided early foundational work through a content analysis of Douyin, critiquing the exposure of children’s privacy and their premature adult-like. Ranzini et al. (2020) later shifted the focus to rights, arguing that “child influencers” are often deprived of legal rights to earnings and safe working conditions. Rafsanjani (2022), employing a normative-juridical framework, found that such participation compromises children’s well-being and that existing legal frameworks are insufficient, reinforcing the need for new regulatory approaches, a call echoed by Ågren’s (2023) suggestion for tailored legislation. To understand the mechanics of this phenomenon, Khojir and Khangheshlaghi (2024) identified four axes structuring child influencers’ virtual lives on Instagram: attracting followers, performing parental roles, virtual identification, and advertising. Most recently, Divon et al. (2025) identified four practices of commodification (e.g., “kids as props”, “transactional childhood”).

### 1.3. Audience Engagement and Feedback in Children’s Digital Identities

The construction of children’s digital identities is not a one-way process; it is dynamically shaped by the interaction between content and its audience. Drawing on digital ethnography and qualitative content analysis of short videos and audience comments on selected hunxue’er accounts, Jiang (2024) found that content featuring Chinese-Caucasian children tends to receive greater visibility and more favorable audience responses. In a related vein, Prasetyo (2025) employed a quantitative survey of 180 Indonesian parents aged 25–45 who use Instagram and TikTok, revealing that parents’ purchasing decisions are substantially influenced by influencer popularity and content appeal. Through focus groups and in-depth interviews, Kołaszewska and Kacprzak (2022) explored the attitudes of both children and parents toward child influencers, finding that while children often expressed admiration, parents exhibited more complex emotional responses and held reservations about purchasing associated products. Similarly, Van den Abeele et al. (2025), through in-depth interviews with 20 followers of “mommy influencers”, found that although these mothers believed featuring children was essential for enhancing the influencers’ credibility, authenticity, and perceived intimacy, they also emphasized employing anti-sharenting strategies to protect their own children’s privacy.

### 1.4. Research Gap and the Contribution of the Present Study

Based on the preceding literature review, existing studies primarily exhibit two research orientations: one is the “risk prevention” paradigm, which systematically reveals the negative consequences of children’s digital exposure on privacy, safety, and psychological well-being; the other is the “identity construction” approach, which delves into how parental media practices shape children’s digital personas. However, the extant literature has the following limitations: In terms of research perspective, there is a lack of theoretical examination of the deeper cultural logic, failing to explain how children’s images are systematically reconstructed into a consumable “entertainment spectacle”. In terms of research focus, although discussions on “sharenting” behaviors are already quite extensive, systematic research focusing on “child influencers” themselves as the core subject remains relatively scarce. In terms of methodological approach, existing achievements mostly concentrate on the analysis of content production, while neglecting the crucial dimension of audience feedback, making it difficult to fully grasp the dynamic interplay mechanisms underlying the phenomenon.

Addressing these limitations, this study innovates in the following three aspects: First, it introduces “media spectacle” theory as the core analytical framework, transcending the existing paradigms of risk and representation to deeply elucidate the cultural logic by which children’s images are commodified and turned into spectacles driven by the attention economy. Second, it explicitly takes “child influencers” as the research object, situating them within the specific context of Chinese short-video platforms to explore the media presentation and identity construction of this group. Third, methodologically, it innovatively integrates computational communication analysis with qualitative textual interpretation, achieving a dual examination of both content production and audience feedback, thereby providing a more comprehensive research perspective for understanding the phenomenon of child influencers on Chinese short-video platforms.

### 1.5. The Present Study

This study draws on the theory of “media spectacle” to construct an analytical framework.

**Figure 1:**
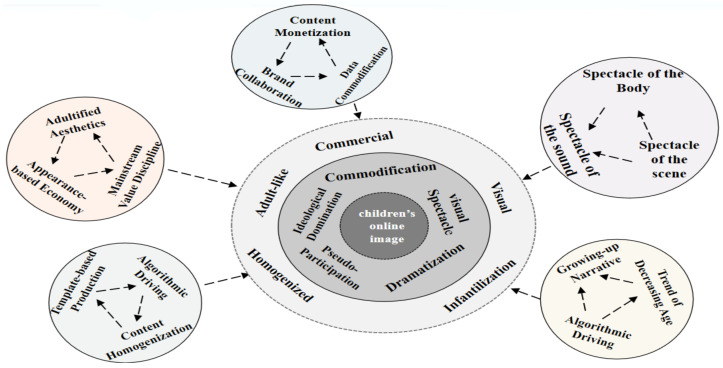
Analytical Framework Diagram from the Perspective of Media Spectacle Theory (Created by the authors).

Based on the theoretical framework of “media spectacle”, this study innovatively combines qualitative research with computational communication methods. Focusing on the construction of children’s digital images on Douyin, it systematically analyzes the underlying “entertainment-oriented” trend (see Figure 1). These theoretical concepts are operationalized as follows: These theoretical concepts are operationalized as follows: dramatization manifests through growing-up narratives and the trend of precocious exposure, packaging children’s developmental trajectories into consumable coming-of-age stories; visual spectacle is embodied in the spectacle of the body, spectacle of the scene, and spectacle of sound, which prioritize sensory stimulation over substantive reflection; commodification operates through content monetization, brand collaboration, and data commercialization, transforming intimate family documentation into commercially driven symbols; ideological domination functions through adultified aesthetics, appearance-based economy, and algorithmic driving, reinforcing mainstream values while disciplining children’s self-presentation; and pseudo-participation is enacted through homogenized content production and algorithmic adaptation, where creators replicate proven formulas under the illusion of creative freedom while subject to implicit platform control. Critically, this pseudo-participation extends to the comment section: viewers’ seemingly spontaneous affective engagement is, in fact, appropriated by platform algorithms and transformed into quantifiable metrics. Simultaneously, ideological reinforcement unfolds subtly within this space—commenters’ expressions of preference often inadvertently consolidate adult-centric values.

Specifically, this study aims to: (1) identify the predominant characteristics in the presentation of children’s images on Douyin within the framework of media spectacle theory; (2) interpret the specific mechanisms by which these five characteristics—namely dramatization, visual spectacle, commodification, ideological domination, and pseudo-participation—manifest in child-centric content; and (3) examine how audience comments function within this spectacular framework. To achieve these goals, the study employs web crawlers to collect extensive data of children’s videos and related user comments, and employing NVivo14 for qualitative text analysis, this research reveals how digital platforms transform childhood experiences into consumable spectacles. Adopting a critical stance toward the platform-driven pan-entertainment of children’s online images, we advocate for safeguarding children’s developmental rights over engagement-centric algorithmic imperatives.

## 2. Materials and Methods

This study adopts a hybrid approach, combining computational tools with content and discourse analysis to explore the “spectacle” construction of children’s social media images. The following sections provide a detailed account of the data collection procedures and analysis techniques used in this study.

### 2.1. Scrapy Crawler Tool

During the data collection phase, we used a high-performance web crawling system based on the Scrapy framework to crawl and analyze data from Douyin 38.2.0 (ByteDance Ltd., Beijing, China). Scrapy framework is an open-source Python 3.12 framework specifically designed for large-scale, structured data extraction from websites (Khder, 2021). The system was designed to minimize its intrusion by implementing dynamic delays (3–8 s) and rotating HTTP request headers. This simulates the browsing behavior of various genuine Douyin users, preventing the system from being identified as a bot. The crawler automatically gathered and stored text, images, and videos that met our predefined research parameters. For instance, this study successfully crawled 500 comments from N1 popular videos, encompassed comprehensive metadata such as user profiles (user_name, user_id), account metrics (followers_count, statuses_count), content features (text, publish-location), and engagement indicators (like-count, comment-count). All references to ‘user behavior’ in this context pertain exclusively to platform users rather than the researchers.

### 2.2. NVivo 14 Software (Lumivero, LLC, Denver, Colorado, USA)

This software is typically used to assist researchers in studying materials such as text, interviews, questionnaires, and images. The coding process was conducted inductively, meaning no a priori coding grid was developed; instead, themes and categories emerged directly from the data. During open coding, extracting raw statements and conceptualizing them into propositions. A total of 21 initial concepts were identified. For instance, comments such as “This kid really doesn’t lie—he says everything out loud” were coded as “Candid remarks” under the initial concept of Authentic Speech. After integrating and sequencing these concepts, 9 preliminary categories were formed. Axial coding is the second level of coding. In contrast to open coding, which focuses on identifying emergent themes, axial coding further refines, aligns, and categorizes the themes (Williams & Moser, 2019). Through in-depth analysis of these 9 preliminary categories, the study distilled 3 main categories. The second author performed the primary coding. To ensure consistency and validity, the analysis was regularly reviewed and discussed with the research team; any interpretive disagreements were resolved through discussion to reach a consensus on the final category structure.

### 2.3. Data Collection

This study employed a purposive sampling approach, selecting the three most-liked (videos ranked in the top three by cumulative like counts within each account’s 2024 output) videos from each of ten targeted accounts (resulting in a total of 30 videos).Videos with high like counts were chosen as they reflect a considerable degree of user recognition and engagement, thereby ensuring that the collected data are both meaningful and representative. The number of accounts was determined based on the principle of theoretical saturation: comments from seven accounts were initially coded and analyzed, after which comments from three additional accounts were incorporated to achieve theoretical saturation, ultimately establishing a final sample of ten accounts. A distributed crawler was used to collect 500 user comments from each of 30 viral videos, yielding a total of 15,000 text entries. Through manual screening, we excluded duplicate comments, comments containing only emojis or stickers, and comments suspected of being generated by bots, so as to ensure that subsequent analyses were based on authentic user feedback. All data were preprocessed and then analyzed using a three-stage coding process (open, axial, and selective) in NVivo 14.

### 2.4. Account Selection

This study investigated prominent child influencer accounts on Douyin, China’s leading short-video platform, which are typically managed by parents. Subject identification was conducted using HuiTu Data 3.9.7 (HuiTu Technology, Hangzhou, China) analytics software, a specialized platform for social media intelligence that provides comprehensive metrics on content performance and audience engagement. Through this tool, we identified relevant accounts using the “#Cute Kids” and “#Parent–Child Interaction” hashtags. To mitigate potential sampling bias inherent in hashtag-based identification, we cross-referenced the identified accounts against Douyin’s recommendation feeds and curated parenting categories, confirming that no major accounts meeting inclusion criteria were systematically excluded by this approach. The selection criteria focused on accounts featuring children under six years old that demonstrated the most significant follower growth during the three-month period leading to mid-December 2024. The following inclusion criteria were applied: accounts had to (1) feature children under six as central protagonists; (2) be parent-managed; (3) demonstrate follower growth during the three months leading to mid-December 2024; (4) have over 200 published videos; and (5) maintain public access. Accounts were excluded if they were brand-verified, primarily featured adult content, or had been suspended during data collection. Based on these criteria, we selected 10 prominent child influencer accounts and their top 3 most-liked videos from each (30 videos total). Among them, there are 6 females and 4 males, with ages ranging from 2 to 6 years old. These children have a large number of followers on social media, with their fan base ranging from 2.1 million to 37.6 million, and the number of their video posts is all above 200. By cross-referencing the accounts’ initial video posting dates with birthday-related video content, we estimated the subjects’ approximate age at their first appearance in digital spaces.

### 2.5. Analytical Framework

To systematically address the research questions, this study adopts a mixed-methods approach, examining the spectacle construction of children’s images from the dual contexts of production and reception. Although the sampling units are videos and the raw data include individual comments, the analytical focus is on identifying the video styles, thematic meanings, and visual language within the text that participate in the construction of the “spectacle”. At the level of production context, to provide an empirical foundation for the qualitative analysis, this study systematically operationalizes three categories of quantitative descriptors: first, account-level indicators (including follower counts, number of videos published, and age at first online appearance), used to characterize the scale of each account’s digital footprint and its developmental context; second, commercial performance indicators (including the number of showcase items, sales volume, and product categories), used to substantiate the commercialization characteristics underpinning the spectacle construction; and third, content type distribution (such as the frequency of entertainment/dance video categories), used to classify and compare video styles. On this basis, prior to comment analysis, the researchers conducted systematic observation and content analysis of the audiovisual content of 30 videos, deeply examining the visual elements, auditory elements, and scene settings to distill the stylistic features and narrative strategies at the level of video production.

At the level of reception analysis, thematic coding of user comments was conducted to reveal the adult-oriented video style characteristics induced by audience preferences. The data from the production and reception levels mutually corroborate each other, collectively revealing the construction mechanisms and core characteristics of child influencers’ online images from the perspective of “media spectacle” theory.

### 2.6. Ethical Considerations

This study analyzed publicly available social media content. The Association of Internet Researchers (AoIR) guidelines indicate that for research focusing on publicly accessible archives where interactions are performative and intended for a public audience, there is a reduced obligation to protect individual privacy (Markham & Buchanan, 2012). Notwithstanding this, we implemented a rigorous ethical protocol that exceeded baseline requirements, given our specific focus on the vulnerability of child subjects. Regarding consent, we sought formal permission from video uploaders (typically parents or guardians) whenever feasible—particularly when contact information was provided—although obtaining individual consent for each comment was not practicable given the scale of the dataset. We firmly uphold a commitment to protecting children’s well-being and agency. To address the vulnerability of the children involved, we avoided quoting comments that could inadvertently identify specific children and excluded any content depicting children in potentially compromising situations, even when such content was publicly available. Furthermore, all visual material was processed using photo-editing tools to remove all personally identifiable information—including names and profile handles—while preserving the contextual and expressive elements essential for our visual analysis. This comprehensive approach, which integrates consent and anonymization, is consistent with our opinion toward sharenting practices.

### 2.7. Researcher Positionality

We approach this study from a hybrid insider-outsider position. As researchers based in China with native proficiency in Mandarin and deep familiarity with Douyin’s platform culture, we possess insider knowledge that enables nuanced interpretation of culturally embedded content and user comments. However, we remain outsiders to the specific community under study, as we are neither parents who engage in sharenting nor content creators themselves. This dual positioning allows us to leverage cultural and linguistic proximity for methodological depth while maintaining critical distance to interrogate the practices and structures we examine. Throughout the research process—from data collection to analysis—we employed reflexive practices, including team debriefings and systematic coding using NVivo, to mitigate potential biases and enhance analytical transparency.

## 3. Results

The basic information of the children selected from 10 accounts in this study is shown in Table 1 (sample numbers N1–N10).

### 3.1. The Visual Mechanism of Spectacle Production

Through the observation and analysis of selected videos, this study identifies three spectacled visual mechanisms that are central to the construction of children’s images in short videos: the spectacle of the body, the spectacle of the scene, and the spectacle of sound.

The dramatic encoding of bodily spectacle constitutes a central feature of contemporary children’s media consumption. Within the logic of consumer society, the presentation of child influencers’ bodies on short-video platforms is highly theatrical. Producers employ multi-layered visual rhetorical strategies to transform children’s bodies into attractive consumer symbols: exaggerated facial expressions and gestures are used to create comic effects, for instance, parents may physically guide a N6’s arms to simulate dance moves, leveraging the child’s lively expressions and movements to attract audiences; unique costumes or role-playing outfits enhance visual impact, as when N6 is dressed in traditional Chinese attire, eliciting comments such as “so cute!”; and cinematic techniques like slow motion and extreme close-ups magnify micro-expressions and bodily gestures during emotional transitions, such as a tightly framed shot of N6 crying with an open mouth, intensifying the video’s dramatic impact. These meticulously choreographed bodily performances not only cater to the audience’s voyeuristic desires but also, on a deeper level, alienate the child’s body into a core productive element within the attention economy—transforming the physical body into a communicable media spectacle.

The scenic spectacle constructs childhood imagery through hyper-real visual environments. Videos often employ filters, special effects, or artificially arranged settings, evoking audiences’ emotional resonance and nostalgic associations with childhood by aesthetically purifying mundane domestic scenes. For instance, a fantastical tableau is created through a manually constructed lotus leaf used as a makeshift shower, depicting N8 bathing with it to craft a fairy-tale-like visual narrative. Auditory spectacle employs layered sound design to achieve emotional manipulation. High-intensity background music, exaggerated sound effects, and carefully paced dialogue work in concert to guide emotional rhythms. Melodic and rhythmic variations in the music establish tonal atmosphere; dramatic audio effects deepen immersion; and controlled speech patterns—pacing and pauses—generate narrative tension. Meanwhile, voiceover narration, frequently delivered by parents as off-screen narrators, deepens empathetic engagement by fostering a sense of direct interaction with the child. For example, when N4 struggles to eat noodles with a spoon, his parent’s off-screen comment, “he doesn’t know where to start”, adds both humor and relatability to the scene.

### 3.2. The Production Mechanism of Precocious Exposure

This study reveals that the trend of featuring increasingly younger children on short video platforms has become a conspicuous media spectacle. In an extreme case, Child N5 (born 4 April 2022) was introduced to an online platform by their parents merely eight days after birth (12 April 2022), with consistent content updates since, enabling audiences to witness the child’s complete developmental trajectory from neonate to toddlerhood. This phenomenon of being exposed online from birth is not an isolated occurrence. Research data indicate that among ten representative cases, a striking 80% of the children (coded N1, N4, N6–N10) were introduced online by their parents before reaching one year of age, and the majority concentrated between two to four months (N1 at approximately four months, N4 and N9 at about two months, and N7 through N10 at around three months). Only 20% of the cases (N2 around three years old, N3 about two years old) began online sharing after their first birthday.

This trend towards younger ages is underpinned by three distinct production mechanisms.

Firstly, content documenting infant development is strategically framed as a high-traffic label by both algorithms and creators due to its perceived “scarcity”. Platforms employ hashtags such as #Human Offspring Evolution to repackage physiological milestones—including a baby’s first roll-over or first taste of solid food—into shareable “content events” Secondly, the “raising-style” account model cultivates emotional attachment by tracking a child’s growth over time, prompting users to develop a sense of “cloud parenting” engagement. Finally, quantitative analysis reveals the synergistic role of algorithmic recommendations and MCNs in driving the mass production of early childhood content. On one hand, platform algorithms demonstrate a clear traffic bias—here referring to the volume of content distribution and user engagement metrics such as views, likes, and shares—toward content emphasizing “infantile characteristics” (such as baby laughter and close-ups). Empirical data indicate that such content generates substantial viewership and engagement; for instance, topics like “human offspring calling mom for the first time” accumulated over 73.25 million views on Douyin, with related videos reaching up to 2.92 million likes. On the other hand, MCNs engage in mass-producing child influencers through standardized scripts (e.g., orchestrating scenarios like “baby’s first time calling mom”).

### 3.3. The Covert Infiltration of Commercial Spectacles

An analysis of sales volume and revenue data from the Douyin product display interface revealed that all sampled accounts featured distinct commercial promotion labels and maintained active product showcases. This finding directly substantiates the commercial nature of their content operations (see Table 2).

Quantitative analysis conducted on our sample suggests that the practice of sharenting—in the cases observed—has been replaced by a commercial model, with all sampled accounts operating product display windows containing 25 to 677 items—exemplified by Account N8’s scaled product shelf of 677 listings. Sales data demonstrate significant polarization: top-performing accounts N1 and N5 achieved 4.61 million and 11.41 million units sold respectively, whereas Account N3 recorded merely 7000 units sold despite listing 399 products, highlighting substantial disparities in commercial conversion efficiency. Category distribution exhibits distinct characteristics: snacks achieve 90% penetration (9/10 accounts) as the primary monetization channel; children’s apparel and maternal-infant products, though covering fewer accounts (2/10), generate the highest single-category sales (4.61 million units for N1); and daily necessities show 70% penetration as supplementary revenue streams. These accounts strategically combine high-frequency but low-priced fast-moving consumer goods with low-frequency but high-profit items, embodying the strategy of “high-frequency low-priced + low-frequency high-value”. Further analysis reveals that account N1 exemplifies a strategy of precision, focusing on a curated cluster of complementary categories—maternal and infant products, apparel, and daily necessities. This precise positioning facilitates high conversion rates, resulting in sales exceeding 4 million units. In stark contrast, account N8 constructs an extensive children’s consumption ecosystem via a massive product assortment of nearly 700 items.

### 3.4. A Typological Analysis of Homogenized Spectacles

Based on the classification framework of video content established in Stephenson et al.’s (2024) “sharenting” research and aligned with the actual categories observed in the ten case studies selected for this paper, the video content from these cases can be categorized into the following four types, demonstrating homogenization. This homogeneity is not coincidental, but rather the result of a multiple parties collaboration among platform economies, algorithmic rules, audience attitude and family content creators (see Table 3).

The content can be classified into four distinct categories: childhood anecdotes (documenting amusing growing-up moments), daily dialogues (parent–child interactions), curated image shares (carefully posed portraits) and entertainment/dance (talent performances). Notably, each account exhibits a clear specialization trend, focusing intensely on only 1–2 content types rather than covering all categories. For instance, accounts such as N2, N7, N8, and N10 predominantly share childhood anecdotes, crafting specific personas through selectively “adorable moments”; N1, N4, N5, and N9 concentrate on daily dialogues, leveraging childish candidness for entertainment; while N6 singularly focuses on dance performances, establishing brand recognition through stylized acts. This typological division reflects content creators’ traffic optimization strategies tailored to platform algorithm rules. Once a particular content style gains higher visibility, creators consistently reinforce it, eventually solidifying into recognizable “persona” templates. Under this mechanism, children’s images are reduced to a set of symbolic typological labels—such as “witty little rascal” “adorably clueless baby” or “gifted prodigy”—which manifest on the Douyin platform in the form of trending topics. These topics systematically aggregate homogeneous content, continually reinforcing users’ cognitive schemas and associative patterns.

### 3.5. The Implicit Influence of Adult-like Spectacles

This study employed a three-level coding process to analyze audience comments as independent analytical units. First, initial concepts expressing similar meanings, pointing to the same phenomenon, or reflecting similar types of audience reactions were clustered into specific categories—for instance, “Candid remarks” and “Disclosure of private matters” were integrated into the category of Authentic Speech. Second, through constant comparison, multiple specific categories sharing a common focus were further summarized into higher-level main categories. This inductive coding logic ultimately yielded a three-level framework comprising 3 main categories, 9 specific categories, and 21 initial concepts. Frequencies were calculated based on the number of comments assigned to each concept, with percentages representing the proportion relative to the total sample. Based on this analysis, the study identifies that the construction of children’s online personas exhibits a distinct characteristic of adult-like orientation, forming a systematic content production framework across three dimensions: personality shaping, image crafting, and emotional appeal (see Table 4).

In the dimension of personality shaping, a marked tension is observed between performances of “Authentic Speech” (6.8%) and “Mature Comprehension” (7.4%). In the former, children’s inadvertent breaches of adult taboos—often through carefully crafted dialogues—unintentionally expose private adult themes, thereby satisfying the audience’s voyeuristic impulses, as seen when N5 discusses marriage with her father. In the latter, children are fashioned into “little philosophers” or “precociously articulate respondents”, using juvenile speech to convey adult-centric values and generating a “contrast-induced endearment” effect—exemplified when N4 cleverly expresses her criteria for a partner by saying, “I wouldn’t want to marry an onion and cry all day”, prompting audiences to marvel, “Sometimes I feel he’s not just a child… he made me see the light”, demonstrating how children’s discourse transmitting adult values evokes cognitive resonance among the audience.

“Image crafting” stands as the most prominent characteristic, accounting for over 70% of total comments. “Adorable Appearance” (30.0%) represents the most frequently mentioned category within this dimension, emphasizing neotenous features, charming outfits, and pleasant vocal qualities that align with cuteness norms, eliciting reactions such as “Her eyes are so big—ship her to me!”. “Likable Behaviors” (9.9%) and “Innocent Kindness” (7.6%) construct an image of childhood purity through motivational speeches and acts of kindness, with audiences noting examples like “How sweet is she! She got startled herself but still went to cover the dog’s ears”. Furthermore, “Unique Talents” (24.7%) complete this crafted image through expressive facial cues and enchanting dance performances, with audiences focusing on children’s performative cooperation and charismatic presentation.

At the level of emotional evocation, two distinctive features resonate strongly with audiences. “Nostalgic Childhood” (4.6%) triggers collective memory through childhood reminiscences and wholesome childlike behaviors, particularly generating comments like “Kids like this are rare nowadays. Back in our time, we’d pick things straight from the ground and eat them without getting sick”. Meanwhile, “Parent–Child Bonding” (9.1%) constructs idealized family connections through heartwarming narratives. Comments archetypizing “the considerate little daughter” who knows to bring water when her mother is tired provide emotional consolation and model ideal family dynamics for audiences. The straightforward comparison in comments such as “Daughters are the best” further reinforces the longing for idealized parent–child relationships.

## 4. Discussion

Based on the analytical perspective of media spectacle theory (Kellner, 2003), this paper explores the entertainment-oriented construction of children’s roles on Douyin. Our study has identified five distinctive characteristics: sensory-oriented imagery, a trend toward younger subjects, commercial motivations, content homogenization, and adultified performance styles. The following sections will detail how these characteristics collectively embody the logic of the spectacle.

### 4.1. From Digital Theater to Algorithmic Prison: The Technological Discipline and Alienation of Child Performance

This study identifies three spectacled visual mechanisms—bodily, scenic, and auditory—that structurally shape children’s performances in short videos. It operationalizes Douglas Kellner’s concept of “visual “, which describes a cultural landscape where sensory stimuli like images and short videos systematically supplant depth and reflection (Kellner, 2003). The identified mechanisms exemplify this prioritization of sensation, functioning as the core technical constituents of a visual spectacle.

On short video platforms, child influencers through exaggerated expressions, costumes, and cinematic techniques such as slow motion and close-ups, while parents leverage their “cuteness” as affective triggers to sustain engagement (Dale et al., 2016). That “cuteness”, such as large forehead, chubby cheeks, big eyes, small nose and mouth, and plump body shape, evokes affective caregiving responses from adults to ensure the survival and nurture of the child, even if the responders are not kin (Glocker et al., 2008). This performativity extends into hyper-real environments constructed with filters and staged settings, transforming homes into perpetual sets and daily life into scripted “scene events”, resonating with McKee’s (2010) concept of narrative scenes. Meanwhile, sound design—through music, effects, and parental voice-overs—orchestrates emotional rhythms to steer audience interpretation, reflecting what H. Huang (2014) called the “adult packaging of childhood”, where auditory layers often overwrite the child’s own voice and reframe their identity through an adult lens.

### 4.2. Datafied Childhood: Intergenerational Exploitation of “Born-to-Be-Influencers”

Our study reveals a significant trend of “early-age” in children’s online presence, with all documented cases involving minors who lack the capacity to understand the long-term consequences of digital exposure or engage with the internet safely (Holloway et al., 2013; Ng Osborn, 2009). This demonstrates how children are systematically incorporated into the digital attention economy before developing full self-awareness. This premature consumption of early childhood epitomizes the “dramatization” logic inherent in media spectacles (Kellner, 2003)—where the developmental journey of infants and toddlers is packaged into consumable “coming-of-age” narratives that deliberately amplify the contrast between innate innocence and performed maturity, thereby heightening dramatic tension and sustaining audience engagement. Empirical evidence from Stephenson et al. (2024) corroborates this trend, showing toddlers and preschoolers as the most prevalent groups in the videos (approximately 37% each), followed by school-aged children (25%).

The algorithmic promotion of topics like “baby’s first time calling mom” on Douyin illustrates how platforms structurally accelerate the early digitalization of children’s identities. This aligns with Fox and Hoy’s (2019) findings on brand-driven sharenting, where platforms transform childhood development into computable data trajectories, reducing children to raw materials in digital production chains—a process underscored by Li and Li’s (2018) observation that big data enables precise mining of private information. Consequently, children are systematically deprived of “digital autonomy” from birth, unlike traditional identity formation that begins with cognitive maturity. As Amon et al. (2022) notes, these children cannot comprehend or consent to their online exposure. These findings underscore the urgent need to establish robust legal and ethical frameworks that prioritize the protection of children’s digital rights over commercial interests and platform engagement.

### 4.3. The Commodification of Childhood Symbols: Image Alienation in the Short-Video Industry

This study reveals that short video platforms function as a catalyst for the commodification of children’s images, wherein the very process of childhood is transformed into a pan-entertainment spectacle. Quantitative analysis of our study confirms that what appears as intimate documentation—covering emotional expression, developmental milestones, and daily routines—is in fact a highly disciplined production system. With individual accounts publishing between 200 to 1062 videos, authentic childhood experiences are systematically reconstructed into consumable entertainment symbols, a phenomenon that directly corresponds to Kellner’s (2003) characterization of media spectacles, in which all content ultimately serves commodification.

This mediated reconstruction of childhood reflects a broader digital trend in which authentic sharing is increasingly supplanted by commercially oriented content, as observed in mommy blogs by Hunter (2016) and Van Cleaf (2015). Within this context, children’s digital identities are strategically crafted to serve parents’ social and financial interests (Jorge et al., 2022), often through fabricated personas distributed via first-person promotional narratives (Ågren, 2023). As Fox and Hoy (2019) note, such commercial sharenting tends to prioritize benefits over privacy, leading parents to consciously trade children’s personal data for social and monetary gain (Barnes, 2006; Abidin, 2017). Similarly, on short video platforms, performative family intimacy is strategically deployed to enhance engagement and embed product promotion, systematically transforming children’s spontaneous behaviors into decontextualized, consumption-driven spectacles (Vizcaíno-Verdú et al., 2022). These practices highlight the pressing need to establish clear regulatory boundaries that protect children’s digital identity and privacy in increasingly commercialized online environments.

### 4.4. Algorithmically Manufactured Childhood: Homogenized Production and the Crisis of Agency

This study reveals a notable trend towards homogenization in children’s short-video content, whose production logic is dictated by the intertwined dynamics of platform algorithms and public attention. This result further exemplifies the characteristic of “Pseudo-Participation”—where users appear to “interact” but are in fact disciplined by algorithms—within the media spectacle (Kellner, 2003). The platform’s traffic-driven incentive mechanisms, for instance, foster the creation of standardized ‘childhood models’, a process that finds its manifestation in Deleuze’s (1995) theory on the digitalization, prediction, and regulation of behavior through algorithms. In pursuit of maximum visibility, creators continually replicate “proven formulas”, reducing the rich diversity of childhood traits—such as spontaneity, charm, or innocence—into a limited set of quantifiable visual labels. Further, Stephenson et al. (2024) indicates that some parents may be posting with the intention of reaching wide audiences. This type of behavior modification has been observed on Twitter, where people who experience a viral event subsequently craft their content to be similar to those in viral posts (Hasan et al., 2022).

From another perspective, parents’ strategies for building social capital—such as deploying follow loops, engagement pods, or leveraging aggregator accounts (Ruiz Gómez, 2024)—further compel the construction of a distinctive influencer identity (Khamis et al., 2017), which often leads to content homogenization (Hudders et al., 2024). In this process, the child’s image is transformed from a multidimensional subject into a mass-reproducible cultural symbol. Such stereotyped representations may adversely shape children’s body image and self-perception (Digennaro, 2024), merging mundane moments with manufactured personas in a manner resembling standardized cultural production. These trends underscore the importance of fostering platform mechanisms that support diverse and authentic forms of expression, rather than reinforcing homogenized digital identities.

### 4.5. The Violence of Cuteness: Structural Oppression in “Performed Childhood”

Short video platforms have established a performative field for childhood performance through algorithmic mechanisms. This performative field not only regulates children’s self-presentation but also systematically reinforces dominant values such as success-driven mentality and the appearance-based economy—core dimensions of what Kellner terms “Ideological Domination” (Kellner, 2003). Within this spectacle, adult aesthetic preferences, amplified and validated by algorithmic incentives, constitute a normative system that increasingly blurs the boundaries between childhood and adulthood. The division between childhood and adulthood is being eroded as children are increasingly exposed to adult information and expected to demonstrate adult competencies (Postman, 1985). This might be because cuteness alone is not enough to create content and sustain attention. Audiences need a story line in the same way that pet influencers and virtual influencers need “wellexecuted character design and a plot (Moustakas et al., 2020). Parent-run baby accounts must craft a digital persona for public consumption infused with real or imagined personality traits and deliver a story line that engages audiences (Ruiz Gómez, 2024).

The role of the audience in this process cannot be overlooked. Our analysis of user comments reveals how audience participation actively influences the performance of childhood. When audiences consistently reward certain behaviors—such as praising “Adorable Appearance” (30.0% of comments) or celebrating “Unique Talents” (24.7% of comments)—they create a feedback that reinforces specific performance norms. This collective validation transforms individual viewing behaviors into a force that disciplines children’s online presentation, making the audience co-facilitator in the spectacle. This dynamic resonates with studies of identity presentation on profile-based platforms—such as social networking sites and personal ads—which have shown that profile creators are highly attuned to audience expectations (Boyd, 2006; Ellison et al., 2006).

This audience-algorithm synergy naturalizes adult-world performance norms as “quality content standards”. The internet’s architecture embeds adult-centric assumptions that render children’s participation either invisible or problematized, privileging adult modes of engagement as the universal standard (Third et al., 2014). In this era of technopoly characterized by capitalist logic and technological mythology, children are prematurely socialized into “docile and obedient lambs” (Ao, 2015). These findings underscore the need to develop child-centered platform governance that resists adult-centric algorithmic values and protects children’s right to authentic self-expression.

## 5. Conclusions

Based on Kellner’s theory of “media spectacle”, this study examines the construction of children’s online personas by “child influencer”—typically managed by parents—on Douyin, identifying five distinctive characteristics that collectively reflect and reinforce the logic of pan-entertainment. These include: (1) Child influencers’ bodies are systematically encoded as visual spectacles through exaggerated expressions, costumes, and cinematic techniques such as slow motion and close-ups, while curated scenic and auditory elements enhance emotional appeal and audience immersion. (2) A pronounced trend toward younger online presence is observed, with 80% of the sampled child accounts appearing before one year of age. (3) The formation of patterns such as “high-frequency low-priced + low-frequency high-value” reflects a distinct commercialization tendency within sharenting practices. (4) Content homogenization manifests through typified templates—childhood anecdotes, daily dialogues, photo collections, and performances, resulting in standardized representations of childhood. (5) The adult-like character of performance styles demonstrates the phenomenon where children’s images are increasingly tailored to adult aesthetic preferences and emotional expectations. This trend is actively reinforced by audience feedback such as likes and comments, collectively forming a “performance–feedback–reperformance” cycle that constructs an adult-oriented narrative paradigm. Our study makes theoretical contributions by extending Kellner’s “media spectacle” theory, revealing how short video platforms construct children as objects of spectacular consumption through a “performance–feedback–reperformance” cycle. Empirically, it systematically demonstrates five key characteristics of the child influencer industry, providing concrete evidence for understanding childhood alienation in the digital era.

### 5.1. Limitations and Future Directions

This study has several limitations. First, regarding sampling bias, the focus on high-growth, high-visibility child influencer accounts with substantial commercial engagement systematically excluded less commercialized and lower-visibility accounts. Consequently, the findings cannot be generalized to the broader population of child content creators across the platform ecosystem. Second, the study did not incorporate direct analysis of platform algorithms or recommendation systems, limiting our understanding of how algorithmic curation interacts with content production and audience reception. Third, the static nature of the data lacks dynamic insight, as the cross-sectional design cannot capture patterns of change over time or the developmental trajectories of individual accounts. Fourth, the cultural specificity of the sample (Chinese Douyin) constrains generalizability to other sociocultural contexts with different regulatory, cultural, and platform environments. Fifth, the observational design precludes causal inferences, as uncontrolled confounding variables (e.g., family environment, parental media literacy, socioeconomic status) may influence observed relationships.

Four research priorities emerge. First, to address sampling bias, future research should adopt stratified sampling that includes accounts across the visibility spectrum—from micro-influencers to non-commercialized amateur accounts—to test whether the identified spectacle mechanisms represent broader patterns.

Second, given the absence of algorithmic analysis, future studies should integrate platform audits to examine how recommendation systems mediate content visibility, revealing the interplay between human production and platform logics.

Third, to overcome static and culturally specific data, longitudinal and cross-cultural designs are needed. Multi-wave tracking would capture how child influencers’ images evolve over time, while cross-platform and cross-national comparisons would test generalizability across different regulatory and cultural contexts.

Fourth, to move beyond observational constraints, future research should employ multi-level modeling that accounts for confounding variables (e.g., parental media literacy, socioeconomic status), disentangling individual, familial, and platform-level factors shaping child influencer outcomes.

### 5.2. Implication

This study makes theoretical contributions by advancing the understanding of childhood alienation within the platform economy through Kellner’s theory of “media spectacle”. It frames the phenomenon not as isolated issues, but as an interconnected system of commodification driven by algorithmic and commercial logics. Theoretically, it extends Kellner’s framework-comprising dramatization, visual spectacle, commodification, ideological domination, and pseudo-participation—by identifying five distinctive characteristics in the construction of children’s digital identities: the sensory-oriented shaping of children’s images, the decreasing age of child subjects online, the commercialization of image-building motives, the homogenization of video content, and the adult-like character of performance styles. These features collectively epitomize and reinforce the pervasive logic of pan-entertainment, whereby childhood is systematically reconfigured into a consumable spectacle. Furthermore, our study uncovers the process of children’s digital identity formation within the platform economy. By analyzing mechanisms such as content homogenization and performance adult-like, we reveal how platform rules establish templates for childhood performance through algorithmic reward systems. This analysis provides a novel theoretical perspective for understanding the construction of digital identities.

On a practical level, this study provides some inspiration for key stakeholders. For parents and educators, it establishes a critical observational lens that helps distinguish performative child content from authentic experiences while strengthening awareness of children’s privacy protection. For the audience of sharenting content, this research fosters critical media literacy by revealing the production mechanisms behind seemingly authentic childhood representations, enabling them to recognize the commercial influences and performance pressures embedded in such content and thus engage with it more reflexively. For platform regulators, the findings urging the establishment of clear consent mechanisms, time protection, and revenue distribution regulations.

## Figures and Tables

**Table 1:** Basic Information of Research Accounts.

No.	Gender	Age	Followers (in Millions)	Video Count	Earliest Video Post Date	Age at First Online Appearance
N1	Female	5 years 1 month	37.607	271	12 March 2020	4 months
N2	Male	5 years 5 months	7.543	525	30 January 2022	3 years
N3	Female	5 years	5.455	504	15 December 2021	2 years
N4	Male	2 years 11 months	2.547	513	7 March 2022	2 months
N5	Female	2 years 8 months	2.149	884	12 April 2022	8 days old
N6	Male	2 years	7.236	330	27 February 2023	2 months
N7	Female	3 years 4 months	4.787	1062	26 November 2021	3 months
N8	Female	4 years	20.770	443	20 March 2021	3 months
N9	Female	6 years 2 months	9.258	909	25 December 2018	2 months
N10	Male	5 years	12.228	779	10 March 2020	3 months

**Table 2:** Sales Performance of Featured Products by Selected Influencers.

No.	Showcase Items	Units Sold	Product Categories
N1	95	4610k+	Maternal & Infant Products, Children’s Apparel, General Merchandise
N2	99	30k+	Snacks
N3	399	7k+	Snacks, General Merchandise
N4	25	30k+	Snacks, General Merchandise
N5	30	11,410k+	Snacks
N6	40	100k+	Snacks
N7	102	60k+	Snacks
N8	677	1200k+	Children’s Apparel, General Merchandise
N9	151	60k+	Snacks
N10	440	2010k+	Snacks, General Merchandise

**Table 3:** Comparative Analysis of Video Categories in case’s Content.

No.	Childhood Anecdotes	Daily Conversations	Photo Collections	Entertainment/Dance
N1	0	237	39	1
N2	233	292	0	0
N3	43	175	324	5
N4	77	436	24	0
N5	91	538	0	255
N6	0	0	0	330
N7	779	227	56	0
N8	373	56	14	0
N9	203	502	204	0
N10	534	229	16	0

**Table 4:** Three-Level Coding Analysis of Video Content Characteristics.

Main Category	Specific Category	Initial Concepts	Representative Comment Count	Percentage of Sample	Example of the Audience Comments
Identity Formation	Authentic Speech	Candid remarks, Disclosure of private matters	379	6.8%	“This kid really doesn’t lie—he says everything out loud, the more he talks, the more we love it”.
	Mature Comprehension	Tactful handling, Philosophic utterance	411	7.4%	“Oh my god, the ‘Do you love Dad or Mom more?’ question is so tricky, but this kid answered it perfectly!” “Sometimes I feel like he’s not just a child. There are things I haven’t even figured out, yet he made me see the light”.
Image Crafting	Adorable Appearance	Cute outfits, Humorous clothing, Childish language, Pleasant voice	1669	30.0%	“Her eyes are so big—ship her to me!” “What’s on her head is so cute!” “Can I say it? He looks like a little gas tank in this outfit!” “Guys, her crying doesn’t sound bad at all—does anyone get it?”
	Likable Behaviors	Motivational speeches, Blessing messages	548	9.9%	“Thank you for your sweetest wishes, sweetie. Your big sister also wishes you endless happiness and a wonderful New Year!”
	Innocent Kindness	Acts of kindness; Kind utterances	420	7.6%	“How sweet is she! She got startled herself, but still went to cover the dog’s ears”.
	Unique Talents	Expressive facial cues; Charming dances	1372	24.7%	“I’m so curious—what’s in front of him? Every time, his expression is so bright, and he cooperates so well!” “Haha, so adorable! Suddenly I realize why—when dancing, his arms and hands stretch out but are too short to reach above his head!”
Sentiment Evocation	Nostalgic Childhood	Childhood reminiscence; Healthy image; Childlike behaviors	255	4.6%	“Kids like this are rare these days. Back in our time, we’d pick things straight from the ground and eat them without getting sick”. “This kid looks so sturdy—definitely the kind grandparents adore!” “I’m dying of laughter—how could he fall asleep with a dried persimmon in his mouth? I thought his lips were swollen!”
	Parent–Child Bonding	Acts of gratitude; Words of appreciation	506	9.1%	“Daughters are the best. When Mom’s tired, she knows to bring water. My rebellious son once said he’d turn into Ultraman and destroy me!”

## Data Availability

The data presented in this study are available on request from the corresponding author.
